# Bilateral Cytomegalovirus Retinitis After Chimeric Antigen Receptor T-cell Therapy for B-cell Lymphoma

**DOI:** 10.7759/cureus.56637

**Published:** 2024-03-21

**Authors:** Leo Meller, Vasan Jagadeesh, Katherine Wilson, Michael C Oca, Timothy Sestak, Nathan Scott

**Affiliations:** 1 Viterbi Family Department of Ophthalmology at the Shiley Eye Institute, University of California San Diego School of Medicine, La Jolla, USA

**Keywords:** cytomegalovirus (cmv), primary diffuse large b-cell lymphoma, car-t, retinitis, cytomegalovirus retinitis

## Abstract

Cytomegalovirus (CMV) retinitis is commonly associated with immunosuppression and can cause irreversible vision loss. Chimeric antigen receptor T-cell (CAR-T) therapy has emerged as an effective cancer treatment option but requires immunosuppression, thereby increasing the possibility of acquiring opportunistic infections such as CMV. We present the case of a 76-year-old female with a history of hypertension and type 2 diabetes mellitus who initially presented with shortness of breath and was diagnosed with the activated B-cell subset of diffuse large B-cell lymphoma (DLBCL). She received multiple cycles of chemotherapy and experienced relapses with cardiac involvement. The patient developed vision loss in the right eye and was diagnosed with bilateral posterior vitritis. She underwent various treatments, including radiotherapy, systemic chemotherapy, cataract extraction, and vitrectomy. After CAR-T therapy, she developed bilateral CMV retinitis, confirmed through polymerase chain reaction testing and managed by valganciclovir. Overall, this case report describes the first reported case of bilateral CMV retinitis following CAR-T therapy for DLBCL. It emphasizes the need for early recognition and treatment of CMV retinitis to prevent permanent vision loss. The report also underscores the importance of regular ocular screening and consideration of prophylactic measures in patients undergoing CAR-T therapy.

## Introduction

Cytomegalovirus (CMV) is a common and opportunistic *Herpesviridae* virus that establishes latency and persists for the entirety of an individual’s life [1]. Globally, it is estimated that the proportion of adults with CMV IgG antibodies is 60-90% [2]. Although it is common to lack overt symptoms with CMV infection, individuals receiving cancer treatment or those who are otherwise immunocompromised are at greater risk of CMV infection causing serious diseases ranging from hepatitis and encephalitis to retinitis [1]. In particular, CMV retinitis is a rare ocular manifestation of CMV infection that causes necrosis of the outer retinal cells and appears as numerous white dots with a granular appearance and varying levels of intraretinal bleeding [3]. As the necrosis worsens, the underlying retinal pigment epithelium atrophies and reveals choroidal vasculature, with serious adverse effects on the visual system including blurry vision, floaters, and loss of peripheral vision [4-5]. Retinal necrosis, hemorrhage, and vasculitis caused by CMV retinitis can further contribute to blindness and even death if the infection remains untreated [5-6].

Recently, chimeric antigen receptor T-cell therapy (CAR-T) has risen quickly as an effective and durable cancer treatment option [7]. CAR-T therapy involves the genetic modification of a patient’s own T-cells to recognize certain blood cancers, including acute lymphoblastic leukemia and diffuse large B-cell lymphoma (DLBCL) [8-9]. Patients receiving CAR-T therapy are at an increased risk of immunosuppression and consequent infection by several opportunistic pathogens such as CMV, Epstein-Barr virus, human herpesvirus 6, and rhinovirus [7,10-12]. Previous research has highlighted the association of CAR-T therapy with adverse ocular outcomes, including acute retinal necrosis, vision changes, and impairment [13]. However, given the relative novelty of CAR-T therapy, a stronger understanding of other potential ocular adverse outcomes is needed. Presently, we report the first case of bilateral CMV retinitis after CAR-T therapy for the treatment of DLBCL and review all existing cases of CMV retinitis associated with CAR-T therapy.

## Case presentation

The patient is a 76-year-old female with a history of hypertension and type 2 diabetes mellitus who initially presented to an outside institution with shortness of breath and was ultimately found to have a pulmonary embolism in 2020. The workup led to a diagnosis of an activated B-cell (ABC) subset of DLBCL. She initially received 5 cycles of rituximab/cyclophosphamide/doxorubicin hydrochloride/vincristine sulfate/prednisone chemotherapy from August 2020 to November 2020, which was complicated by a pericardial effusion and pulseless electrical activity arrest. In January 2021, she developed facial swelling and was diagnosed with relapsed lymphoma, for which she received six cycles of polatuzumab/bendamustine/rituximab from April to July 2021.

One year later, in July 2022, she relapsed, now with a mediastinal mass causing mediastinal/esophageal compression and concern for cardiac invasion. She also began experiencing blurry vision in the right eye at this time, prompting a referral to ophthalmology. Upon initial ophthalmological evaluation, three weeks after the initial onset of vision loss, her visual acuity was 20/32 OD and 20/50-1 OS. She was noted to have bilateral posterior vitritis without clear evidence of retinitis, which was thought to be highly suspicious for intraocular lymphoma. A biopsy was considered but foregone given the patient’s medical instability, her known history of lymphoma, and her exam findings and imaging suggestive of bilateral metastasis to the vitreous.

Since the patient was already receiving external beam radiotherapy (EBRT) for intracranial lesions, the choice was made to proceed with EBRT to both eyes rather than intravitreal methotrexate to treat these suspected vitreous metastases. She was also started on systemic chemotherapy complemented by the iR2 regimen (lenalidomide 20 mg, ibrutinib 560 mg at home, and rituximab infusions). The patient was subsequently referred to our ocular oncology service for evaluation. At the time of our initial evaluation in August 2022, she had already begun bilateral EBRT treatments as well as systemic chemotherapy. The examination was notable for dense cataracts in both eyes and no evidence of active intraocular inflammation. She did report some worsening of vision after starting EBRT, though these symptoms were consistent with exam findings of worsening cataracts. As there was no evidence of active ocular disease, she was sent back to her uveitis provider and underwent cataract extraction with intraocular lens implantation (CE/IOL), starting with the left eye, without complications.

Around the time of her one-month post-operative visit, the patient was referred by her oncology team to the bone marrow transplant service for consideration of CAR-T therapy due to her favorable response to iR2. She began CAR-T in early November 2022. She then underwent CE/IOL of the right eye in December 2022, again without intraoperative complications. However, she was noted to have possible choroidal detachments with vitreous hemorrhage on her post-operative week two visit. She underwent a pars plana vitrectomy with choroidal drainage and subsequent resolution of the choroidal detachments. Later that month, she completed CAR-T treatment without neurological events or complications, and her highest cytokine release syndrome (CRS) grade was two.

**Figure 1:**
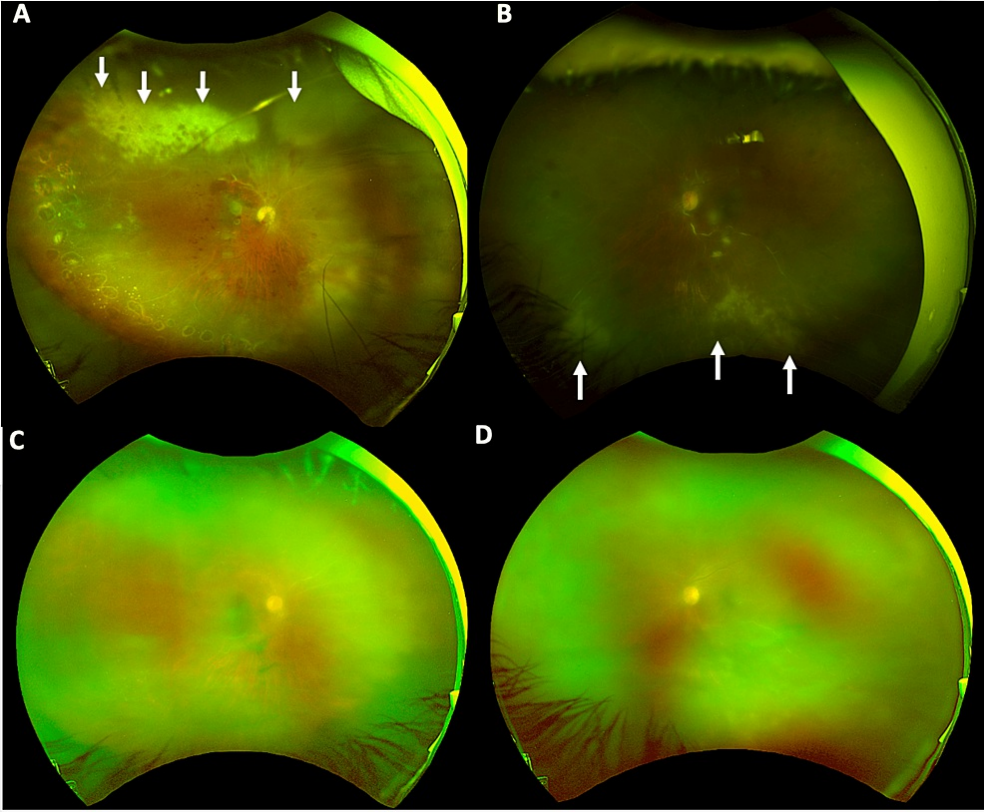
Fundus photography of the right and left eyes In the right eye (A), there is a slightly hazy view secondary to active vitritis; the nerve is pale with juxtapapillary hemorrhages; the macula is flat, but the foveal reflex is blunted, and there is extensive intraretinal hemorrhage. The vasculature appears sclerotic, particularly over the superior arcade. There is evidence of prior laser, from pars plana vitrectomy with endolaser to treat choroidal detachments, in the temporal and inferior quadrants. There is active retinitis from 10:00 to 1:30 (white arrows). The left eye (B) has similar findings to the right; however, the sclerotic vasculature and retinitis are in the inferior quadrant (white arrows). (C-D) Pre-cataract surgery imaging (C: right eye, D: left eye)

Unfortunately, in March 2023, she had recurrent vitreous hemorrhage that required a second pars plana vitrectomy, at which time she was noted to have areas concerning retinitis (Figure 1). She was started on valganciclovir 900 mg BID, and an anterior chamber tap was performed for both eyes. PCR studies returned positive for CMV in both eyes. In July 2023, her vision was 20/80 OS and 20/200 OD. Unfortunately, the patient ultimately passed away due to refractory stage IV ABC-subtype DLBCL.

## Discussion

CAR-T therapy has shown immense promise for treating relapsed and chemotherapy-resistant blood cancers [14]. However, it can increase the risk of immunosuppression in patients, as they are less capable of mounting robust CD4 and CD8+ T-cell responses that are typically activated with viral infection [14]. This enables the virus to replicate and spread more readily [15], making patients more vulnerable to infection, especially by opportunistic infections such as CMV [16]. To the best of our knowledge, we report the first case of simultaneous bilateral CMV retinitis following CAR-T therapy for DLBCL. Currently, only two other cases of CMV retinitis following CAR-T therapy have been reported (Table 1), and in both cases, the infection was unilateral. In all cases, patients presented with symptoms of decreased visual acuity. Further examination also revealed other notable pathologies, such as macula edema, choroidal and retinal detachment, and retinal necrosis. Importantly, across all cases, patients underwent CAR-T therapy after several rounds of other cancer treatments (i.e., chemotherapy). Not only are these patients immunocompromised by their disease state, but their previous treatments have also increased their vulnerability to infections like CMV. In our present case, the patient had multiple comorbidities (i.e., type 2 diabetes mellitus, hypertension) that are known to damage the endothelium and vasculature of the eye [17], which puts her at great risk for CMV infection of the eye.

**Table 1:** Literature review of other cases of CMV retinitis after CAR-T therapy CAR-T: chimeric antigen receptor therapy, CMV: cytomegalovirus, DLBCL: diffuse large B-cell lymphoma, IV: intravenous, IVIG: intravenous immunoglobulin, KP: keratic precipitates

Case	Authors, year	Age, sex	CAR-T indication	Time to CMV retinitis	Laterality	Spread of CMV	Treatment	Outcome
1	Present case	76, F	Relapsed DLBCL	3 months	Bilateral	No	Oral valganciclovir	Patient deceased of DLBCL
2	Bin Dokhi et al., 2022 [29]	43, F	Primary refractory DLBCL	n/a	Left	No	Intravitreal ganciclovir injections; oral valganciclovir; IV foscarne; CMV IVIG	Regained 20/20 vision
3	Zu et al., 2022 [30]	58, M	Multiple myeloma	4 months	Right	No	IV ganciclovir; oral ganciclovir; virectomy with silicone tamponade	Regained 20/1000 vision

Although CMV in the eye primarily targets vascular endothelial cells, it may also infect retinal pigment epithelial cells, ultimately causing retinal necrosis [18]. Following this, patients may report blurry vision, which can progress to blindness if necrosis persists or the patient develops a retinal detachment [19]. Thus, CMV retinitis can be a vision-threatening infection without early recognition and intervention. Intravenous ganciclovir and/or oral valganciclovir are common treatments for patients diagnosed with CMV [20]. Valganciclovir has been seen as an effective and safe prophylactic treatment for CMV in cases of kidney [21], pancreas [22], and liver transplants [23]. Valganciclovir has also been used to reduce CMV in patients with glioblastomas receiving temozolomide and radiation therapy [24]. In pediatric populations receiving CAR-T therapy, acyclovir is recommended as a prophylactic if they are found to have a positive baseline serology for herpes simplex virus and varicella zoster virus [25]. However, there is no published data on anti-viral prophylaxis for CMV in pediatric or adult populations on CAR-T therapy [25]. Valganciclovir is a modification of acyclovir with greater bioavailability, and currently, its efficacy for CMV prophylaxis is variable [26]. Nonetheless, further study into the utility of Valganciclovir for preventing and managing CMV infection and for use as a prophylactic in patients undergoing CAR-T therapy is warranted. Valganciclovir is contraindicated in patients with granulocytopenia or neutropenia, as well as in patients with decreased bone marrow activity, all of which may be seen in patients undergoing CAR-T therapy [14,27]. These contraindications could explain why its use in CAR-T patients has not been explored yet. The current lack of valganciclovir usage for CMV prophylaxis in CAR-T therapy patients could be related to the cost of the drug, which can be nearly $200 for patients to pay out of pocket; however, prior studies have shown valganciclovir as a prophylactic measure to be safe and cost-effective [21]. While CMV retinitis is an infrequent manifestation of CMV in some immunocompromised populations (i.e., transplant patients), CMV retinitis accounts for up to 85% of CMV infections in patients with AIDS and is the leading cause of blindness and vision loss among this population [20]. Given the potential adverse outcomes associated with CMV retinitis, providers should closely monitor patients undergoing CAR-T therapy for CMV and consider prescribing some type of prophylaxis for potential CMV retinitis and other manifestations of the infection for patients particularly at risk. Lastly, ophthalmologists must remain vigilant when treating patients with CAR-T therapy, and measures such as regular dilated fundoscopic examinations should be taken to screen patients for CMV infection to avoid potential complications and improve patient prognosis [28].

## Conclusions

We report the first case of bilateral CMV retinitis after CAR-T therapy for DLBCL. Given the adverse ocular outcomes and retinal necrosis associated with CMV retinitis, timely recognition and treatment are critical to prevent permanent vision loss. Our case underscores the importance of regular ocular screening for potential infection in patients undergoing CAR-T therapy and encourages the consideration of prescribing prophylactic valganciclovir against potential CMV retinitis and other infections in patients receiving this treatment.
